# Neutralising antibodies against the Omicron variant after COVID-19 vaccination in UK haemodialysis patients

**DOI:** 10.1016/S0140-6736(22)00104-0

**Published:** 2022-01-20

**Authors:** Edward J Carr, Mary Wu, Ruth Harvey, Roseanne E Billany, Emma C Wall, Gavin Kelly, Haemodialysis COVID-19 consortium, Crick COVID Immunity Pipeline, Michael Howell, George Kassiotis, Charles Swanton, Sonia Gandhi, David LV Bauer, Matthew PM Graham-Brown, Rachel B Jones, Rona M Smith, Stephen McAdoo, Michelle Willicombe, Rupert Beale

**Affiliations:** 1 The Francis Crick Institute, London, UK; 2 Worldwide Influenza Centre, The Francis Crick Institute, London, UK; 3 Department of Cardiovascular Sciences, University of Leicester, Department of Renal Medicine, University Hospitals of Leicester NHS Trust, UK and NIHR Leicester Biomedical Research Centre, Glenfield Hospital, Leicester, UK; 4 Genotype-to-Phenotype UK National Virology Consortium (G2P-UK); 5 Department of Medicine, University of Cambridge School of Clinical Medicine, Cambridge, UK; 6 Cambridge University Hospitals NHS Foundation Trust, Hills Road, Cambridge, UK; 7 Centre for Inflammatory Disease, Department of Immunology and Inflammation, Faculty of Medicine, Imperial College London, UK; 8 Renal and Transplant Centre, Imperial College Healthcare NHS Trust, Hammersmith Hospital, London, UK; 9 UCL Dept of Renal Medicine, Royal Free Hospital, London, UK

The SARS-CoV-2 variant-of-concern (VOC) B.1.1.529 Omicron is now the predominant VOC in the UK ^1^. The burden of >30 mutations in Omicron spike suggests at least a degree of vaccine evasion ^2^, and UK Health Security Agency estimates of vaccine efficacy against infection are reduced compared to Delta ^1^. The critical question is how well existing vaccines will protect clinically extremely vulnerable groups against infection. The COVID-19 death rate for in-centre haemodialysis (IC-HD) patients during the Delta wave was 14.65 (95% CI 11.49-18.67) per 1,000 patient years, the highest rate for any OpenSAFELY-defined comorbidity ^3^. The increased transmissibility of Omicron is likely to prove challenging in haemodialysis (HD) units, where in-unit transmission with prior VOCs has occurred ^4^. We therefore sought to determine the neutralising antibody (nAb) titres in IC-HD patients, a cohort we have previously shown to have attenuated nAb responses to Delta ^5^.

In the UK, the IC-HD vaccination schedule in place is complex. The majority of IC-HD patients are considered ‘fully vaccinated’ after two doses, and ‘boosted’ after three. ‘Boosting’ eligibility criteria were finalised on 14 September 2021 ^6^. A subset of IC-HD patients, due to their use of additional immunosuppression (for example for failed renal transplants) or other comorbidities, are eligible for a three dose primary course (announced 1 September 2021) ^7^. These patients are already permitted a fourth ‘booster’ dose 3 months after their third dose.

To assess the induction, maintenance and diversity of nAbs we convened UK-wide consortium study assessing **n** eutralising **a** ntibody after C **O** VID vaccination in hae **m** od **i** alysis patients (NAOMI) ^5^. This is an observational multi-centre meta-cohort study to compare neutralising antibody responses between different vaccine regimens, and in pre-specified patient subgroups. Previously, we compared neutralising antibody responses after two doses of the adenoviral vector Oxford/AstraZeneca vaccine (ChAdOx-1, AZD1222) or the Pfizer-BioNTech mRNA vaccine (BNT162b2). mRNA vaccine neutralising responses against wildtype virus and variants of concern (VOCs) were similar to those seen in healthcare or laboratory workers ^5,8,9^.

Here we report the first nAb titres (nAbT) against Omicron in the at-risk IC-HD population (n=98) at 158 days [146-163] after dose 2, and at 27 days [21-35] after dose 3 (median, [interquartile range, IQR]). We used live virus microneutralisation assays as previously ^5^, and report Delta as a comparator VOC, with full demographics listed in the appendix (appendix p2). Doses 1 and 2 were either AZD1222 (n=30) or BNT162b2 (n=68). All third doses were BNT162b2 (at full dose). Earlier timepoints from one HD centre have already been reported ^5^. Given the urgency of these data, we locked this first set once n>=50 sera after dose 3 were available for testing. These patients were vaccinated in September - November 2021 and are from two UK HD centres (appendix p2), reflecting the local variation in the deployment of third doses.

Firstly, we assessed the nAbT against Omicron and Delta at a median of 158 days after two doses of either AZD1222 or BNT162b2 (appendix p3). After two doses of AZD1222 in IC-HD patients, the median nAbT against either VOC was less than the lower limit of detection of our assay (<1:40), in keeping with our previous report of Delta nAbT at 1 month after the second dose ^5^ (Omicron IQR <40-<40, Delta IQR <40-110). At 158 days after two doses of BNT162b2, median nAbT against Delta were 112 (IQR <40-342) and median Omicron nAbT were below the range of the assay (appendix p4, IQR <40-157).

Next, we considered the effect of an additional full dose of BNT162b2 (n=50, appendix p4). For AZD1222-AZD1222-BNT162b2 (AZD-AZD-BNT, n=21) recipients, Delta titres rose after a third dose to a median nAbT of 282 (IQR <40-1250). A third dose provided a statistically significant increase in the proportion of AZD-AZD-BNT patients with Delta or Omicron nAbT>40 (McNemar’s test, *P*=0.023, 0.0077 respectively). However, the median titres against Omicron remained below the quantitative range (<40, IQR <40-270). Recipients of BNT162b2-BNT162b2-BNT162b2 (BNT-BNT-BNT, n=29) had boosted nAbTs against Delta from 112 to 461 (4.1x fold change, IQR 171-1214), and developed detectable nAbTs against Omicron at a median nAbT of 236 (IQR <40-603) after their third dose. A third dose provided a statistically significant increase in the proportion of BNT-BNT-BNT patients with Delta or Omicron nAbT>40 (McNemar’s test, *P*=0.0077, 0.0094 respectively).

For both AZD-AZD-BNT and BNT-BNT-BNT recipients there are a proportion of IC-HD patients that do not mount nAbT responses to either VOC (AZD-AZD-BNT IC_50_<40 after dose 3: 33% [n=7] and 52% [n=11] of 21 patients against Delta or Omicron respectively; BNT-BNT-BNT 3% [n=1] and 28% [n=8] of 29 patients respectively). We hypothesised that these may be patients taking immunosuppressants or with immunosuppressive comorbidities – beyond the immunosuppression associated with end-stage renal disease and haemodialysis itself – and therefore stratified our analysis by the presence of or absence of an immunosuppressed state (appendix p2). The immunosuppressed AZD-AZD-BNT patients had a median nAbT against Omicron below the lower limit of the assay (n=5, IQR <40-<40) and immunosuppressed BNT-BNT-BNT patients had a median nAbT against Omicron of 135 (~50% of the response of the rest of the cohort [n=6, IQR <40-491]). In the non-immunosuppressed recipients, the median Omicron nAbT after dose 3 was 107 for AZD-AZD-BNT recipients (n=16, IQR <40-578) and 236 for BNT-BNT-BNT recipients (n=23, IQR 66.6-777).

The main limitation of our study is its observational nature, risking unbalanced groups for comparisons. For example, the AZD-AZD-BNT and BNT-BNT-BNT cohorts are matched imperfectly, with AZD1222 recipients being older (mean age [standard deviation]: 68.8 [11.2] vs 60.3 [12.1] respectively, t-test *P*=0.001). Therefore, we have reported responses within these vaccine cohorts, not between. We temper this limitation by the importance of these data: IC-HD patients are at increased risk of severe disease or death ^3^, and our data help to inform strategies to mitigate that risk over the coming weeks and months. Vaccine-induced cellular responses are likely to contribute to the protective effect of vaccination. T cell responses to Omicron-spike derived peptides appear comparable to peptide pools from other VOCs in healthy individuals ^10^. In infection-naïve IC-HD patients, after two doses of either vaccine, a reduction in T cell responsiveness to peptides of ancestral S1 and S2 has been reported ^11^. Together, these suggest that Omicron infection in previously vaccinated HD patients might result in diminished cellular responses compared to healthy individuals.

In summary, we report the first nAbT against Omicron in IC-HD, a highly vulnerable population to COVID-19, frequently requiring hospitalisation and with an excess risk of death. Homologous vaccination with BNT-BNT-BNT generated quantifiable nAbT against Delta or Omicron in the majority of IC-HD patients. Heterologous vaccination with AZD-AZD-BNT in IC-HD patients, provides quantifiable nAbT against Delta in >50% of IC-HD patients, but not against Omicron, where >50% of IC-HD patients nAbT were below the quantifiable range. A substantial fraction of IC-HD patients would appear to remain at risk from Omicron infection.

There are several implications of these data. Firstly, the deployment of third doses took ~ 8 weeks between eligibility announcements for third/booster doses and their receipt in this highly vulnerable patient group. This contrasts with their very rapid access to first doses ^5^. Secondly, a lack of a quantifiable response (‘non-response’) after two doses does not predict on-going ‘non-response’ to third doses. We suggest that each further dose reduces this fraction further. Some of these ‘non-responders’ are already eligible for four doses in the UK, as their primary course has already been deemed 3 doses due to immunosuppression use, or co-morbidities ^7^. Thirdly, that adequate nAb titres against Delta in IC-HD required three doses of vaccine, and this is reflected in the epidemiological data from the Delta wave in IC-HD ^3^. Finally, Omicron neutralisation will require at least three vaccine doses, perhaps four doses, in UK IC-HD patients, particularly as the kinetics of waning of Omicron nAb are unknown. Together our data shows that the current generation vaccines still have utility in CEV patient groups, and that the number of doses that constitute an appropriate primary course differs between VOCs: for Omicron, three doses in IC-HD may be insufficient.

CS reports grants from AstraZeneca, BMS, Boehringer Ingelheim, Invitae (formerly Archer Dx), Ono-Pharmaceuticals, Pfizer, and Roche-Ventana, unrelated to this Correspondence; personal fees from Bicycle Therapeutics, Genentech, GRAIL, Medicxi, Metabomed, Roche Innovation Centre Shanghai, Sarah Canon Research Institute, Amgen, AstraZeneca, BMS, Illumina, GlaxoSmithKline, MSD, and Roche-Ventana, unrelated to this Correspondence; and stock options from Apogen Biotech, Epic Biosciences, GRAIL, and Achilles Therapeutics, unrelated to this Correspondence. DLVB reports a grant from AstraZeneca, unrelated to this Correspondence. RBJ reports participation in a GlaxoSmithKline advisory board, unrelated to this Correspondence. RMS is the chief investigator for PROTECT-V, a basket trial of prophylactic interventions in multiple at-risk patient groups including haemodialysis patients, supported by research funding from Union Therapeutics, NIHR, GSK, LifeArc, Kidney Research UK and Addenbrooke’s Charitable Trust. All other authors declare no competing interests. DLVB and RB are members of the Genotype-to-Phenotype UK National Virology Consortium. Funding details and acknowledgments can be found in the appendix. All data (anonymised) and full R code to produce all figures and statistical analysis presented in this Correspondence are available online on GitHub (https://github.com/EdjCarr/Crick-HD-Omicron-2021-12).

## Supplementary Material

Appendix
