# Supplementary material for: Neutralising antibodies against the Omicron variant after COVID-19 vaccination in UK haemodialysis patients
Source: Lancet. Author manuscript; Available in PMC 2022 Mar 2. (PMC8776276; doi:10.1016/S0140-6736(22)00104-0)
Supplement: Appendix [file EMS141049-supplement-Appendix.pdf]

<u>Appendix for</u>

Neutralising antibodies against the Omicron variant after COVID-19 vaccination in UK haemodialysis patients

Edward J Carr[1*], Mary Wu[1*], Ruth Harvey[2], Roseanne E Billany[3], Emma C Wall[1], Gavin Kelly[1], Haemodialysis COVID-19 consortium, Crick COVID Immunity Pipeline[1], Michael Howell[1], George Kassiotis[1], Charles Swanton[1], Sonia Gandhi[1], David LV Bauer[1,4], Matthew PM Graham-Brown[3], Rachel B Jones[5,6], Rona M Smith[5,6], Stephen McAdoo[7,8], Michelle Willicombe[7,8], Rupert Beale[1, 4 & 9]

Affiliations

1 The Francis Crick Institute, London, UK
2 Worldwide Influenza Centre, The Francis Crick Institute, London, UK
3 Department of Cardiovascular Sciences, University of Leicester, Department of Renal Medicine, University Hospitals of Leicester NHS Trust, UK and NIHR Leicester Biomedical Research Centre, Glenfield Hospital, Leicester, UK
4 Genotype-to-Phenotype UK National Virology Consortium (G2P-UK)
5 Department of Medicine, University of Cambridge School of Clinical Medicine, Cambridge, UK
6 Cambridge University Hospitals NHS Foundation Trust, Hills Road, Cambridge, UK
7 Centre for Inflammatory Disease, Department of Immunology and Inflammation, Faculty of Medicine, Imperial College London, UK
8 Renal and Transplant Centre, Imperial College Healthcare NHS Trust, Hammersmith Hospital, London, UK
9 UCL Dept of Renal Medicine, Royal Free Hospital, London, UK

Contents

Table 1
Figure 1
Methods
Author contributions
Acknowledgements
Supplementary References
Consortium membership

	AZD1222	BNT162b2
	n = 30	n = 68
Age (years)		
	68.8 (11.2)	60.3 (12.1)
Gender		
Female	13 (43.3%)	25 (36.8%)
Male	17 (56.7%)	43 (63.2%)
Vaccine		
AZD1222	30 (100%)	0 (0%)
BNT162b2	0 (0%)	68 (100%)
Dialysis Centre		
Cambridge	16 (53.3%)	6 (8.8%)
Leicester	14 (46.7%)	62 (91.2%)
Additionally immunosuppressed		
No	24 (80%)	61 (89.7%)
Yes	6 (20%)	7 (10.3%)

Table 1 - Demographics of the third dose haemodialysis cohort

IC-HD cohort stratified by the formulation of their first two vaccines. Immunosuppression defined as previously [1]. Age is shown as mean (standard deviation).

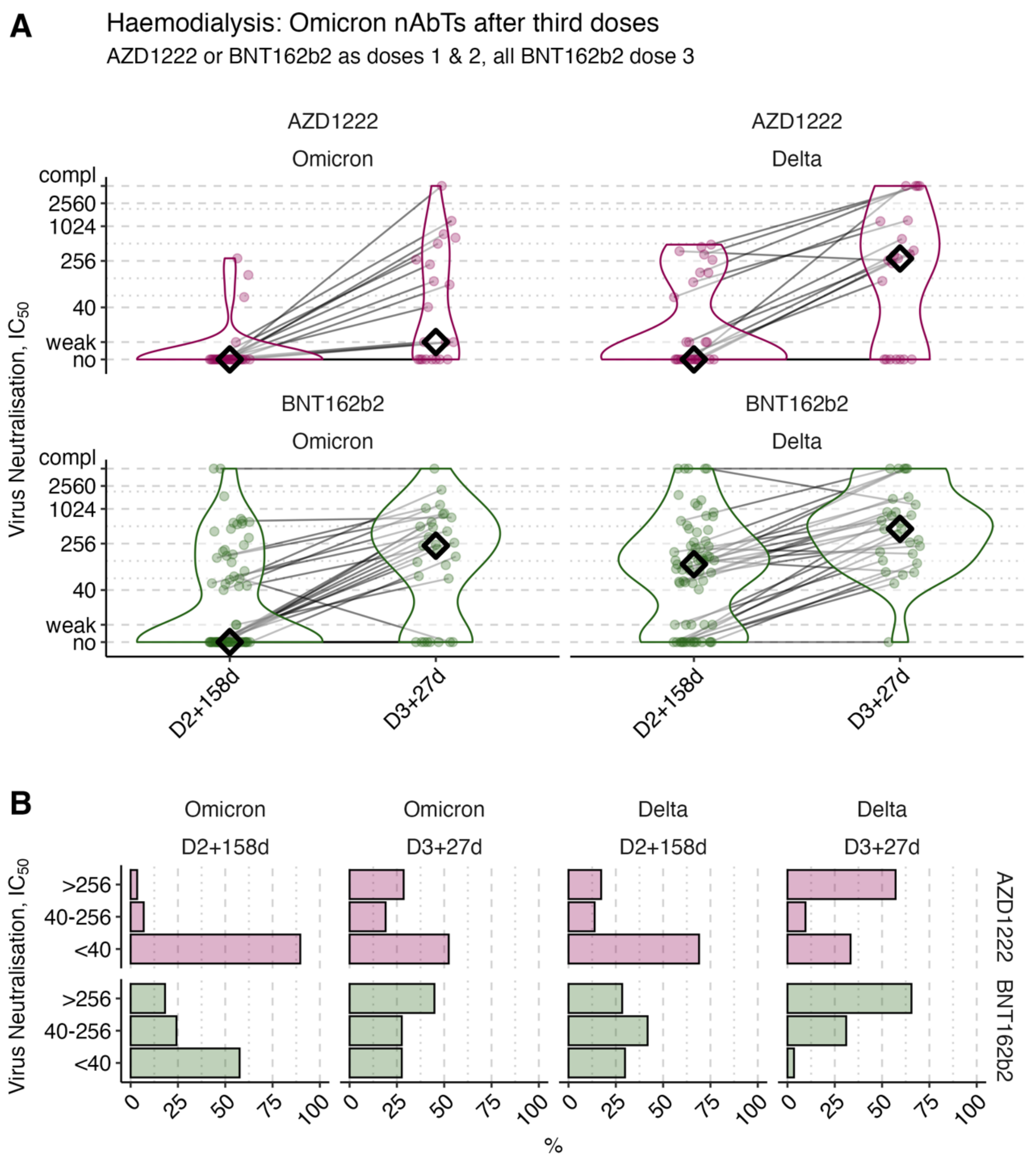

Figure 1 – The neutralising antibody titres against Omicron and Delta SARS-CoV-2 VOCs before and after third doses

(A) Live virus microneutralisation titres for either the Omicron or Delta SARS-CoV-2 variants-of-concern, reported as IC_{50}, are shown at 158 days [IQR 146-163, n=97] after the second dose (D2+158) and 27 days [IQR 21-35, n=50] after dose 3 (D3+27). Each individual is a point, and samples from the same individual are joined by a line (21 paired AZ samples; 28 paired BNT samples). No, weak and complete (compl) inhibition are shown, with gridlines to indicate IC_{50} of 64,128,512 and 2048. Median IC_{50} are marked by a black diamond.

(B) The percentages of IC50s after stratification in low (<40), medium (40-256) or high (>250) neutralising antibody titres are shown at the dates in (A) for both Delta and Omicron. after two doses of either AZD1222 or BNT162b2 and after a subsequent dose of BNT162b2. In (A)-(B), recipients of doses 1 and 2 AZD1222 or BNT162b2 are plotted in purple or green respectively.

Methods

Study objectives and design

We are performing a cohort study of 1,200 IC-HD patients across the UK [2]. The study has several objectives:

1. Confirm the immunogenicity of BNT162b2 and AZD1222 in IC-HD patients, including the generation of neutralising antibodies.
    a. Confirm augmentation of the antibody response with the second dose of vaccine
    b. Assess the longevity of the antibody response, including neutralising antibody.
2. Compare the profiles of neutralising antibodies generated between BNT1262b and AZD1222 IC-HD recipients.
3. Compare the profiles of neutralising antibodies generated by either vaccine between different age groups, different genders, different ethnicities, and different primary renal diseases.
4. Compare the profiles of neutralising antibodies generated between patients with and without diabetes or with and without immunosuppression.
5. Exploratory / discovery phase, where novel patterns / correlations are identified to provide hypothesis for testing in other cohorts / specifically targeted studies.

For any cohort comparison we expect, given the nature of the UK's IC-HD population (its ethnicities, the frequencies of diabetes, immunosuppression) to be able to assemble groups of >100 patients for each comparison.

We planned serum collections were before vaccination, 28 days after each vaccination, and 6 & 12 months after commencing vaccination.

The study design is pragmatic and serum collection dates are reviewed in light of changing vaccination policy.

Clinical cohorts

Two haemodialysis centres are used in this report. In centre haemodialysis patients were included if they were able to consent into their local study and were clinically

eligible to receive the available vaccine. Home haemodialysis patients and peritoneal dialysis patients were not included. The data shown is censored for individuals who received two doses of vaccine, and had available sera ~28 days after their third dose. Anonymised (coded only against a research identifier) sera and phenotype data were provided for central analysis: age, gender, ethnicity, diabetes, immunosuppression, primary renal disease, alongside the dates of vaccine, vaccine manufacturer and the dates of serum sampling. Ethnicity was recorded as Asian, Black, Mixed, White or Other (in line with UK government advice at the time of commencing the study https://webarchive.nationalarchives.gov.uk/20210224165417/https://design-system.service.gov.uk/patterns/ethnic-group/). Diabetes was recorded as Y/N, and we defined immunosuppression as Y/N as in Billany et al. [1]. Individuals were vaccinated intramuscularly as part of their usual care, with either 0.5mL [not less than 2.5×10^8 infectious units] AZD-1222, ChAdOx1-S [recombinant] (Oxford-AstraZeneca) or 30ug BNT162b2 (Pfizer-BioNTech), at the interval indicated in Figure 1.

Leicester cohort

Patient samples were collected as part of the study “PHENOTYPING SEROCONVERSION FOLLOWING VACCINATION AGAINST COVID-19 IN PATIENTS ON HAEMODIALYSIS”, with REC approval from (West Midlands - Solihull Research Ethics Committee, REC: 21/WM/0031) sponsored by the University of Leicester and included consent for samples to transfer to the Francis Crick Institute. This work was conducted locally with support from the NIHR Leicester Biomedical Research Centre and funding from the Leicester Hospitals Charity, University Hospitals of Leicester NHS Trust. Data from these patients have been published previously [1–3].

Cambridge cohort

This prospective observational cohort study, “SARS CoV-2 antibody responses in immunocompromised patients” includes patients recruited from the Department of Nephrology, at Cambridge University Hospitals NHS Foundation Trust (East Midlands- Leicester Central Research Ethics Committee: REC 20/EM/0180). The study is sponsored by Cambridge University Hospitals NHS Foundation Trust.

Consent was obtained as part of the study to allow for anonymised blood samples to analysed by academic and/or industry collaborators, both within and outside the UK. This work was funded by Addenbrooke's Charitable Trust, Vasculitis UK and supported by the Cambridge University Biomedical Research Centre.

Serological Analysis and live-virus neutralisation

All serum samples were collected during routine IC-HD sessions from the HD circuit, without additional venepuncture. Sera were separated from blood in local laboratories and stored frozen. Sera were shipped to the Crick on dry ice, and barcoded whilst frozen. All live-virus microneutralisation were performed as described previously [3].

Virus variants

The SARS-CoV-2 B.1.1.7 isolate ("Alpha") was hCoV-19/England/204690005/2020, which carries the D614G, Δ69-70, Δ144, N501Y, A570D, P681H, T716I, S982A and D1118H mutations in Spike,3 and was obtained from Public Health England (PHE), UK, through Prof. Wendy Barclay, Imperial College London, London, UK through the Genotype-to-Phenotype National Virology Consortium (G2P-UK). The B.1.617.2 ("Delta") isolate was MS066352H (GISAID accession number EPI_ISL_1731019), which carries the T19R, K77R, G142D, Δ156-157/R158G, A222V, L452R, T478K, D614G, P681R, D950N mutations in Spike, and was kindly provided by Prof. Wendy Barclay, Imperial College London, London, UK through the Genotype-to-Phenotype National Virology Consortium (G2P-UK). The BA.1 ("Omicron") isolate was M21021166, which carries the A67V, Δ69-70, T95I, Δ142-144, Y145D, Δ211, L212I, G339D, S371L, S373P, S375F, K417N, N440K, G446S, S477N, T478K, E484A, Q493R, G496S, Q498R, N501Y, Y505H, T547K, D614G, H655Y, N679K, P681H, A701V, N764K, D796Y, N856K, Q954H, N969K, and L981F mutations in Spike, and was kindly provided by Prof. Gavin Screaton, University of Oxford, Oxford, UK through the Genotype-to-Phenotype National Virology Consortium (G2P-UK).

Data analysis, statistics

Data analysis was performed in R4.0.4/Rstudio. Anonymised data wrangling used a mix of base R and tidyverse. As previously [2–4], IC_{50} values above the quantitative limit of detection of the assay (>2560) were re-coded as 5120; IC_{50} values below the quantitative limit of the assay (< 40) but within the qualitative range were re-coded as 10 and data below the qualitative range (i.e. no response observed) were re-coded as 5. The resulting IC_{50} values are winsorized (40-2560). To assess responses to third doses in either AZD-AZD-BNT or BNT-BNT-BNT groups, McNemar's χ^2 test was performed on 2x2 contingency tables, where columns were categorised nAbT (<40 or >40) before dose 3, and rows were categorised nAbT (<40 or >40) after dose 3. IC_{50} values are shown on a log2 scale throughout. Plots were generated using ggplot2 and ggpubr packages.

Data Sharing

All R code to reproduce all figures and analyses is freely available at (https://github.com/EdjCarr/Crick-HD-Omicron-2021-12/). The public dataset omits dialysis centre, age, gender and dates, to maintain anonymity.

Ethics

This work is covered by the following REC approvals: REC: 21/WM/0031 and REC 20/EM/0180.

Role of the funding source

This work was supported by Kidney Research UK, NKF, PKD charity, Kidney Wales and several Kidney Patient Associations [Exeter, North Staffs and South Cheshire, Northamptonshire, South Eastern and Wessex], the MRC and core funding from the Francis Crick Institute, which receives its funding from Cancer Research UK, the UK Medical Research Council, and the Wellcome Trust. The funders of the study had no role in study design, data collection, data analysis, data interpretation, or writing of

the report. The corresponding authors had full access to all the data and the final responsibility to submit for publication.

Contributors Statement

Edward J Carr - Investigation, data curation, data analysis, Writing - original draft, review & editing. Has access to and has verified underlying data
Mary Wu - Investigation, Methodology, Resources, Writing – review & editing, Conceptualization
Roseanne Billany - Investigation, Data Curation, Resources.
Ruth Harvey - Investigation, Methodology, Resources, Writing – review & editing, Conceptualization
Emma C Wall - Investigation, Data Curation, Resources
Gavin Kelly - Formal Analysis, Validation
Haemodialysis COVID-19 consortium - Investigation, Resources
Crick COVID Immunity Pipeline - Investigation, Data Curation, Resources
Michael Howell - Project administration, Supervision, Writing – review & editing, Conceptualization
George Kassiotis - Writing – review & editing, Conceptualization
Charles Swanton - Supervision, Funding acquisition, Project administration, Writing – review & editing, Conceptualization
Sonia Gandhi - Supervision, Funding acquisition, Methodology, Project administration, Writing – review & editing, Conceptualization. Has access to & has verified underlying data.
David LV Bauer - Methodology, Formal Analysis, Visualization, Writing – review & editing, Conceptualization.
Rona Smith - Supervision, Resources, Conceptualization
Rachel B Jones - Supervision, Resources, Conceptualization
Matthew Graham-Brown - Supervision, Resources, Conceptualization
Stephen McAdoo - Conceptualization, Supervision, Funding acquisition
Michelle Willicombe - Conceptualization, Supervision, Funding acquisition
Rupert Beale - Supervision, Funding acquisition, Project administration, Writing – review & editing, Conceptualization, has access to & has verified underlying data.

Acknowledgements

We thank the patients, their families, and the dialysis and renal unit staff who have delivered this at the frontline of a fast-moving pandemic, and the responsive rapid funding from Kidney Research UK and partners.

This research was funded in whole, or in part, by the Wellcome Trust [FC011104, FC011233, FC001030, FC001159, FC001827, FC001078, FC001099, FC001169]. The Genotype-to-Phenotype National Virology Consortium (G2P-UK) is supported by UK Research and Innovation and the UK Medical Research Council [MR/W005611/1], and by the Francis Crick Institute which receives its core funding from Cancer Research UK [FC011104], the UK Medical Research Council [FC011104], and the Wellcome Trust [FC011104]. For the purpose of Open Access, the author has applied a CC BY public copyright licence to any Author Accepted Manuscript version arising from this submission. The authors would like to thank all the study participants, the dialysis and renal unit staff, the staff of the NIHR Clinical Research Facility at UCLH including Kirsty Adams and Marivic Ricamara. We would like to thank Jules Marczak, Gita Mistry, Nicola Bex, Simon Caiden, Bobbi Clayton, Beatriz Montaner and the staff of the Scientific Technology Platforms (STPs) and COVID-19 testing pipeline at the Francis Crick Institute. We thank Prof. Wendy Barclay of Imperial College and the wider G2P-UK consortium for the Alpha and Delta strains used in this study, and Max Whiteley and Thushan I de Silva at The University of Sheffield and Sheffield Teaching Hospitals NHS Foundation Trust for providing source material. We thank Prof. Gavin Screaton of the University of Oxford for the Omicron strain used in this study. We thank Dr Laura McCoy of UCL for the CR3009 antibody. We also thank Marg Crawford, Robert Goldstone, Jerome Nicod, and Harshil Patel for generation and processing of sequencing data. We acknowledge support from the NIHR Leicester Biomedical Research Centre and the Cambridge University Biomedical Research Centre. EJC acknowledges the support of Health Education East of England, in particular Drs Francesca Crawley, Sarah Fluck, Lisa Willcocks and John Firth, to facilitate his out-of-programme experience. This work was supported by the Francis Crick Institute which receives its core funding from Cancer Research UK [FC011104, FC011233, FC001030, FC001159, FC001827, FC001078, FC001099, FC001169], the UK Medical Research Council

[FC011104, FC011233, FC001030, FC001159, FC001827, FC001078, FC001099, FC001169], and the Wellcome Trust [FC011104, FC011233, FC001030, FC001159, FC001827, FC001078, FC001099, FC001169] and funded by the MRC [MRC-Pandemic Award-PRJ-20009 to RB, EJC] and Kidney Research UK [SP/VACCINE/2021 to MW, SM, EJC & RB], with KRUK supported by the NKF, the PKD charity, Kidney Wales and several Kidney Patient Associations [Exeter, North Staffs and South Cheshire, Northamptonshire, South Eastern and Wessex], and the Royal Free Charity, the Leicester Hospitals Charity, Oxford Hospitals Charity, and Addenbrooke's Charitable Trust.

Supplementary references

1Billany RE, Selvaskandan H, Adenwalla SF, *et al.* Seroprevalence of antibody to S1 spike protein following vaccination against COVID-19 in patients receiving hemodialysis: a call to arms. *Kidney Int* 2021; **99**: 1492–4.

2Carr EJ, Wu M, Harvey R, *et al.* Neutralising antibodies after COVID-19 vaccination in UK haemodialysis patients. *Lancet* 2021; **398**: 1038–41.

3Wall EC, Wu M, Harvey R, *et al.* Neutralising antibody activity against SARS-CoV-2 VOCs B.1.617.2 and B.1.351 by BNT162b2 vaccination. *Lancet* 2021; **397**: 2331–3.

4Wall EC, Wu M, Harvey R, *et al.* AZD1222-induced neutralising antibody activity against SARS-CoV-2 Delta VOC. *Lancet* 2021; : S0140-6736(21)01462-8.

Haemodialysis COVID-19 consortium

Sherna F Adenwalla[1], Paul Bird[1], Christopher Holmes[1], Katherine L Hull[1], Daniel S March[1], Haresh Selvaskandan[1], Jorge Jesus-Silva[1], Julian W Tang[1], Joanna Hester[2], Fadi Issa[2], Martin Barnardo[3], Peter Friend[3], Andrew Davenport[4], Catriona Goodlad[4], Vignesh Gopalan[4], Theerasak Tangwonglert[4], Hans J Stauss[5], Alex G Richter[6], Adam F Cunningham[6], Marisol Perez-Toledo[6], Gemma D Banham[7], Nadya Wall[8], Candice L Clarke[9,10], Maria Prendecki[9,10], Joseph Beckett[3], Katherine Bull[11], Sushma Shankar[3], Scott Henderson[4], Reza Motallebzadeh[4,12], Alan D Salama[4], Lorraine Harper[7,8], Patrick B Mark[13,14], Sian Griffins[15], Soma Moran[15], Rita Ng[16], Claire Freeman[16], Nicholas R Pritchard[16], Jacinta Lee[17], Vivian Yiu[18], Anil Chalisey[16], Hannah Stacey[16]

1 Department of Cardiovascular Sciences, University of Leicester and Departments of Renal Medicine and Clinical Virology, University Hospitals of Leicester, UK
2 Transplantation Research & Immunology Group, Nuffield Department of Surgical Sciences, University of Oxford, UK
3 Oxford Transplant Centre, Nuffield Department of Surgical Sciences, University of Oxford, UK
4 UCL Dept of Renal Medicine, Royal Free Hospital, London, UK
5 Institute of Immunity & Transplantation, Royal Free Hospital, London, UK
6 Institute of Immunology and Immunotherapy, College of Medical and Dental Sciences, University of Birmingham and UHB NHS Foundation Trust, UK
7 Department of Nephrology, University Hospitals Birmingham NHS Foundation Trust, Birmingham, UK
8 Institute Applied Health Research, College of Medical and Dental Sciences, University of Birmingham and UHB NHS Foundation Trust, Birmingham, UK
9 Centre for Inflammatory Disease, Department of Immunology and Inflammation, Faculty of Medicine, Imperial College London, UK
10 Renal and Transplant Centre, Imperial College Healthcare NHS Trust, Hammersmith Hospital, London, UK
11 Nuffield Department of Medicine, University of Oxford, UK
12 Research Department of Surgical Biotechnology, Division of Surgery and Interventional Science, University College London, UK
13 Glasgow Renal and Transplant Unit, Queen Elizabeth University Hospital, Glasgow, UK
14 Institute of Cardiovascular and Medical Sciences, University of Glasgow, UK
15 Wales Kidney Research Unit, Division of Infection and Immunity, Cardiff University School of Medicine, Heath Park, Cardiff, UK
16 Cambridge University Hospitals NHS Foundation Trust, Hills Road, Cambridge, UK
17 Department of Medicine, University of Cambridge School of Clinical Medicine, Cambridge, UK
18 West Suffolk NHS Foundation Trust, Hardwick Lane, Bury St Edmunds, Suffolk, UK

Crick COVID Immunity Pipeline consortium membership list

Bobbi Clayton
Sina Namjou
Vanessa Silva
Meghan Poulten
Philip Bawumia
Murad Miah
Samuel Sade
Mauro Miranda
Tom Taylor
Ilenia D'Angelo
Mercedes Cabrera Jarana
Mahbubur Rahman
Janet Abreu
Sandeep Sandhar
Neil Bailey
Simon Caidan
Marie Caulfield
Mary Wu
Ruth Harvey
Lorin Adams
Caitlin Kavanagh
Scott Warchal
Chelsea Sawyer
Mike Gavrielides
Jag Kandasamy
Karen Ambrose
Amy Strange
Titilayo Abiola
Nicola O'Reilly
Philip Hobson
Ana Agau-Doce
Emma Russell
Andrew Riddell
Svend Kjaer
Annabel Borg
Chloë Roustan
Christophe Queval
Rachel Ulferts
Steve Gamblin

All The Francis Crick Institute, London, UK
